# Effects of different fermentation temperatures on microbiomes of cigar tobacco leaves

**DOI:** 10.3389/fbioe.2025.1550383

**Published:** 2025-02-25

**Authors:** Yun Jia, Sida Guo, Wanrong Hu, Qianying Zhang, Yue Wang, Zhengcheng Zhang, Zhishun Chai, Dongliang Li

**Affiliations:** ^1^ China Tobacco Technology Innovation Center for Cigar, China Tobacco Sichuan Industrial Co., Ltd., Chengdu, Sichuan, China; ^2^ Industry Efficient Utilization to Domestic Cigar Tobacco Key Laboratory of Sichuan Province, China Tobacco Sichuan Industrial Co., Ltd., Shifang, Sichuan, China

**Keywords:** cigar tobacco leaves, fermentation temperature, physicochemical metabolites, microbial community structure, microbial functions

## Abstract

**Introduction:**

Microbiomes of cigar tobacco leaves play a pivotal role during the fermentation, and fermentation temperature is a key factor in shaping the structure and function of the microbial community. This study aimed to investigate the effects of different temperatures (30°C, 35°C, 40°C, 45°C, and 50°C) on the microbiomes of cigar tobacco leaves, providing insights into the complex interactions among temperature, microbes, and physicochemical metabolites.

**Methods:**

Firstly, the physicochemical metabolites of cigar tobacco leaves under various fermentation temperatures were detected by gas chromatography-mass spectrometry. Subsequently, the impacts of different temperatures on microbial biomass and community structure were revealed by quantitative real-time PCR and amplicon sequencing, and the biomarkers at different fermentation temperatures were identified by LEfSe analysis. Finally, the functional potential of microbes was predicted by correlation analysis.

**Results:**

The bacterial biomass increased initially and peaked at 8.4 × 10^9^ copies/g at 35°C, then decreased as the temperature rose. The fungal biomass exhibited a downward trend with increasing temperature, reaching a maximum of 3.9 × 10^6^ copies/g at 30°C. When the fermentation temperature exceeded 45°C, the growth of both bacteria and fungi was significantly restricted. Amplicon sequencing results indicated that *Staphylococcus* and *Aspergillus* genera dominated the bacterial and fungal communities, respectively. As the temperature increased, the relative abundance of *Staphylococcus* decreased first and then increased (46.1%–98.5%), while that of *Aspergillus* increased first and then decreased (34.9%–77.4%). Additionally, correlation analysis suggested that microbial communities shaped by different temperatures were responsible for the differences in physicochemical metabolites of cigar leaves. The biomarkers identified in the low-temperature fermentation group, including *Staphylococcus*, *Stemphylium*, *Sampaiozyma*, and *Filobasidium*, were likely responsible for the production of flavor metabolites, the accumulation of sugars, and the elevated ratio of potassium ions to chloride ions contents. Biomarkers in medium and high-temperature fermentation groups, such as *Aspergillus*, *Neodymella*, *Acinetobacter*, *Pelomonas*, *Brevundimonas*, and *Alkalihalobacillus*, might contribute to the degradation of nitrogen-containing substances and alkaloids.

**Discussion:**

This study revealed the unique microbial community structure shaped at different temperatures and its potential correlation with physicochemical metabolites. These findings will help to further optimize the fermentation process of cigar tobacco leaves and develop functional microorganisms suitable for different fermentation temperatures.

## 1 Introduction

Cigars are products made by rolling tobacco leaves, consisting of wrapper, binder, and filler (Frankenburg, 1950). The production process of cigars mainly includes cultivation, drying, fermentation, rolling, and aging. Among them, fermentation is a complex biochemical process that not only determines the final flavor of tobacco leaves but is also essential for reducing bitterness and enhancing combustibility (Li et al., 2020; Zheng et al., 2022). Traditionally, the fermentation process begins with adjusting the initial moisture content to approximately 30%, followed by fermentation under natural or artificially controlled conditions of temperature (35°C–45°C) and relative humidity (75%–85%). During this process, tobacco leaves need to be turned periodically to exclude impurities, ensure homogeneity, and prevent local overheating (Di Giacomo et al., 2007).

Previous studies have demonstrated the presence of abundant microbial populations in tobacco leaves and their fermentation environments (Chattopadhyay et al., 2021; Joshi et al., 2024). These microorganisms play diverse roles in tobacco fermentation, including the degradation of macromolecules (e.g., protein, starch, cellulose), the production of flavor components (e.g., Dihydroactinidiolide, Megastigmatrienone), and the degradation of harmful substances (e.g., nicotine) (Wang et al., 2024; Xue et al., 2023). However, the specific functions of these complex microbial communities in particular environments remain unclear. Recently, the applications of multi-omics technologies (such as amplicon sequencing, metagenomics, and metabolomics) and related statistical methods have provided new possibilities for in-depth exploration of microbial community functions in fermentation ecosystems (Hall et al., 2018). For instance, Wang et al. revealed the microbial communities in cigar tobacco leaves from China, the Dominican Republic, and Indonesia by high-throughput sequencing, and found that *Bacillus*, *Vibrio*, and *Sphingomonas* were closely related to flavor production (Wang et al., 2024).

Fermentation temperature, as an important environmental factor affecting the growth and metabolism of microorganisms, has a significant impact on the structure and function of the microbial community in fermented products. In previous studies, single-factor experiments and uniform design were used to investigate the effects of fermentation temperature, relative humidity, initial moisture content, and fermentation time on the quality of different varieties of cigar tobacco leaves. The results indicated that the fermentation temperature had a greater impact on the content of flavor components than other fermentation factors (Jia et al., 2023b). During the stack fermentation of cigar tobacco leaves, the temperature of the stack rose from ambient temperature of 28°C–46.5°C, and then gradually decreased and stabilized around 40°C (Guo et al., 2021). Ren et al. also investigated the effects of fermentation chamber temperatures (20°C, 27°C, and 34°C) on the microbial community of tobacco leaves, finding that the richness and diversity of bacterial communities initially increase and then decrease with rising temperature, reaching the highest at 27°C (Ren et al., 2023). However, due to the gap between the chamber temperature and the actual temperature within the tobacco stack, these research results may not accurately reflect the direct impact of temperature on the microbial community and physicochemical metabolites. Furthermore, different types of tobacco leaves require different fermentation temperatures due to variations in origin, year, variety, grade, and maturity. For example, Sichuan Dexue No. One and Hubei Shiyan No. One cigar varieties were optimally fermentation at 27°C and 45°C, respectively, to enhance their quality (Li et al., 2020; Ren et al., 2023).

In summary, the fermentation temperature plays a pivotal role in shaping the microbial community and determining the quality of cigar tobacco leaves. However, it remains unclear how fermentation temperature regulates the composition and function of the microbial community in cigars, as well as how these alterations affect the quality of the tobacco leaves. Therefore, according to experience and literature, five temperature points of 30°C, 35°C, 40°C, 45°C and 50°C were set to study the effects of temperature on physicochemical metabolites and microbial communities in cigar leaves. Through correlation analysis, this study unveiled the interplay between microorganisms and the physicochemical metabolites of cigar leaves. The results would guide the optimization of fermentation conditions and the development of functional microbial agents, thereby improving the quality and characteristics of cigar tobacco leaves.

## 2 Materials and methods

### 2.1 Materials and reagents

In this study, the filler of Dexue one was used as the research object, supplied by China Tobacco Sichuan Industry Co., Ltd. (Sichuan, China). A single-factor experiment design was employed to examine the influence of different temperatures on the physicochemical metabolites and microbial communities of cigar leaves. The detailed fermentation process was performed as follows, 2 kg of tobacco leaves were moistened with distilled water to achieve a moisture content of 30%. Subsequently, the leaves were evenly stacked in five constant temperature and humidity incubators with different temperature settings, each maintaining the relative humidity of 75%. The temperatures were set to 30°C, 35°C, 40°C, 45°C, and 50°C, with three biological replicates for each condition. Following a 30-day fermentation period, the samples were harvested and preserved at −80°C for subsequent analysis of the microbial communities and physicochemical metabolites.

The reagents for High-Performance Liquid Chromatography (HPLC) were sourced from Thermo Fisher Scientific (Waltham, MA, United States). Phenylethyl acetate was purchased from Sigma-Aldrich Co. Ltd. (St. Louis, MO, United States). The QuEChERS extraction kit, which includes 4 g MgSO_4_, 1 g NaCl, 1 g NaCitrate, 0.5 g disodium citrate sesquihydrate, and 50 mL tubes with ceramic homogenizers, was purchased from Agilent (Santa Clara, CA, United States). SYBR was obtained from Novazan (Nanjing, China). The E.Z.N.A. (easy nucleic acid isolation) soil DNA Kit was purchased from Omega (Omega Bio-tek, Norcross, GA, United States).

### 2.2 Physicochemical composition detection

The contents of reducing sugar (RS), total sugar (TS), total alkaloids (NIC), total nitrogen (TN), potassium ion (K), and chloride ion (Cl) were detected by an active pharmaceutical ingredient continuous flow instrument (Futura -II, Alliance, FR) (Liu et al., 2021). According to Jia et al., metabolite components were extracted using acetonitrile and QuEChERS extraction kit (containing 4 g MgSO_4_, 1 g NaCl, 1 g NaCitrate, 0.5 g disodium citrate sesquihydrate), and then detected by gas chromatography-mass spectrometry (GC-MS). The separation of compounds was carried out on the DB-5MS column (60 m × 0.25 mm × 1.0 μm, Agilent Technology, Santa Clara, CA, United States). The GC oven program started at an initial temperature of 60°C, increased to 250°C at a rate of 2 °C/min, and then was held at 250°C for 20 min before ramping up to 290°C at 5 °C/min. Helium was used as the carrier gas at the flow rate of 1.2 mL/min. The MS spectra were operated in the electron impact mode with an ion source temperature of 230°C and an ionization voltage of 70 eV. The mass scan range was 26–400 amu with a scanning rate of 0.2 scan/s (Jia et al., 2023a). Qualitative analysis of compounds was performed by matching the mass spectra with the National Institute of Standards and Technology and Wiley Library databases. Compound quantification was calculated based on the ratio of the peak area of a specific compound to that of the phenylethyl acetate internal standard.

### 2.3 Quantitative real-time PCR

To quantify the microbial biomass in the samples, quantitative real-time PCR (qPCR) was performed on a CFX Connect™ Real-Time system (Bio-Rad, Hercules, CA, United States). Cigar leaves were ground into powder with liquid nitrogen, and genomic DNA was subsequently extracted with the soil DNA kit. The purity, concentration, and integrity of extracted DNA were assessed by the NanoDrop 2000 (Thermo Scientific, Waltham, MA, United States) and gel electrophoresis. Primers 341 F (5′-CCTAYGGGRBGCASCAG-3′) and 806 R (5′-GGACTACHVGGGTWTCTAAT-3′) were used for bacterial biomass analysis, and primers ITS1F (5′-CTT​GGT​CAT​TTA​GAG​GAA​GTA​A-3′) and AFP308 (5′-CGA​ATT​AAC​GCG​AGT​CCC​AAC-3′) were used for fungal biomass analysis. Each reaction was performed in 10 μL reaction mixtures containing 5 μL of SYBR mix, 0.2 μL of ROX Reference Dye, 0.4 μL of each primer (10 μmol/L), and 1 μL of DNA template. The qPCR thermocycling steps were set as follows: 95°C for 3 min, followed by 40 cycles of 95°C for 5 s, 58°C for 30 s, and 72°C for 60 s. For the construction of standard curve, the template DNA with known copy number was diluted to various concentrations and added to the PCR reaction system as the DNA template. Each point on the calibration curve was measured in triplicate. The gene copy number was calculated by comparing the threshold cycle (Ct) value with the standard curve. The R^2^ values for the calibration curves of bacteria and fungi were 0.9996 and 0.9994, respectively (Supplementary Figure S1).

### 2.4 Amplicon sequencing and analysis

Cigar leaves were ground to powder with liquid nitrogen, and genomic DNA was subsequently extracted with the soil DNA kit. The purity, concentration, and integrity of extracted DNA were measured by the NanoDrop 2000 and gel electrophoresis. The V3-V4 region of bacterial 16S rRNA genes was amplified by primers 27 F (5′-AGRGTTYGATYMTGGCTCAG-3′) and 1492 R (5′-RGYTACCTTGTTACGACTT-3′). The fungal internal transcribed spacer 1 (ITS1) region was amplified by primers ITS1F (5′-CTT​GGT​CAT​TTA​GAG​GAA​GTA​A-3′) and ITS4R (5′-TCC​TCC​GCT​TAT​TGA​TAT​GC-3′) (Jia et al., 2024). The PCR products were purified using the AMPure^®^ PB beads (Pacific Biosciences, CA, United States) and quantified with Qubit 4.0 (Thermo Fisher Scientific, United States). Then, sequencing was performed by Pacbio Sequel IIe System (Pacific Biosciences, CA, United States) by Majorbio Bio-Pharm Technology Co. Ltd. (Shanghai, China). UPARSE (version 7.1) was used to cluster the sequencing results into operational taxonomic units (OTUs) based on 97% sequence similarity. Sequences annotated as chloroplast or mitochondrial were removed from all samples. The OTUs sequences were annotated using the RDP classifier (version 2.11) against the Silva bacterial database and the Unite fungal database with a confidence threshold of 70%.

### 2.5 Sensory quality evaluation

After fermentation, the tobacco leaves were rolled into cigarettes with a length of 110 mm and a diameter of 14 mm. These cigarettes were then stored for 1 month in an incubator with a controlled temperature of 20°C and a relative humidity of 60%. The sensory quality evaluation was conducted using the Standard Evaluation Form provided by Great Wall Cigar Factory (Sichuan, China) (Hu et al., 2022). The quality characteristics consisted of mellowness, richness, matureness, irritation, smoothness, fluentness, sweetness, cleanliness, aftertaste, combustibility, ash color, and ash coagulation. For the evaluation, a panel of 10 well-trained assessors was assembled to evaluate the sensory quality of the cigarettes.

### 2.6 Statistical analysis

Principal-coordinate analysis (PCoA) was performed using the ade4 and ggplot2 packages in R (version 3.5.1). The linear discriminant analysis (LDA) effect size (LEfSe) algorithm was used to identify the representative bacterial and fungal taxa at different fermentation temperatures. Spearman’s pairwise correlations and the significance of the correlations were calculated using the *corr*. test function and the psych package in R (version 3.5.1). Significance difference analysis and Z-score normalization were conducted using SPSS (version 22.0, SPSS Inc., Chicago, IL, United States). Further statistical analysis and graphics were performed in Microsoft Excel (Microsoft Office, Redmond, WA, United States) and GraphPad Prism Software (version 8.0, GraphPad Software, San Diego, CA, United States).

## 3 Results

### 3.1 Comparison of chemical indexes

**FIGURE 1:**
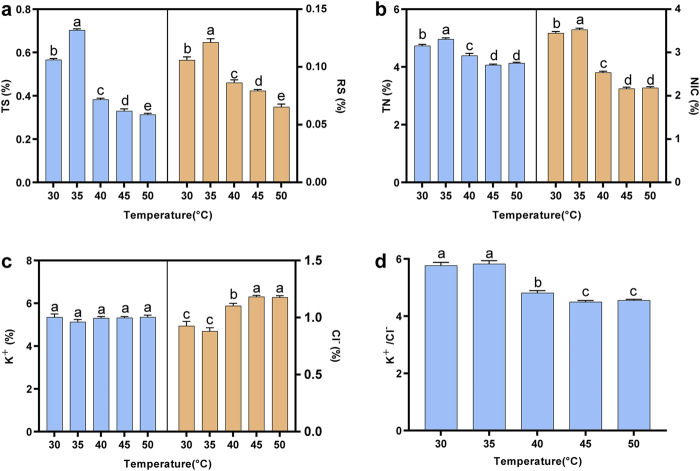
The contents of chemical indexes in tobacco leaves fermented at different fermentation temperatures. **(A)** Total sugar content and reducing sugar contents; **(B)** total nitrogen content and alkaloids contents; **(C)** potassium ion and chloride ion contents; **(D)** the ratio of potassium ion to chloride ion content. TS: total sugar; RS: reducing sugar; TN: total nitrogen; NIC: alkaloid. Letters indicated significant differences based on Tukey’s HSD.

In this study, the contents of reducing sugar (RS), total sugar (TS), total alkaloids (NIC), total nitrogen (TN), potassium ion (K), and chloride ion (Cl) in cigar tobacco leaves fermented at different temperatures were detected. The contents of total sugar and reducing sugar are important indicators of tobacco smoking quality, as sugar compounds could form a variety of flavor substances after the Maillard reaction (Arihara et al., 2017). As shown in Figure 1A, the contents of total sugar and reducing sugar initially increased with rising fermentation temperature, peaked at 35°C (0.70% for total sugar and 0.12% for reducing sugar), and then gradually decreased. Conversely, high-temperature fermentation promoted the Maillard reaction, leading to a decrease in sugar content compared to lower temperatures (Alsafra et al., 2023). Meanwhile, the sensory evaluation results suggested that low-temperature fermentation was more conducive to the improvement of sweetness, which was consistent with chemical indexes (Supplementary Table S1).

Proteins produce a burnt feather flavor when heated, which directly affects the irritancy of tobacco leaves (Liu et al., 2021). A high content of alkaloids not only affects human health but also contributes to the bitterness, astringency, and irritancy of tobacco leaves (Kaminski et al., 2020). Figure 1B showed that the contents of total nitrogen and alkaloid increased initially and then decreased with increasing temperature. When the fermentation temperature reached 45°C or 50°C, the contents reached their lowest levels, with total nitrogen at 4.1% and alkaloids at 2.2%, significantly lower than the contents observed in the other temperature treatments. The results indicated that high-temperature fermentation was more beneficial in improving the irritancy and cleanliness of tobacco leaves (Supplementary Table S1). This might be because high-temperature fermentation could enhance enzyme activity, leading to the degradation of proteins and alkaloids into small molecules such as amino acids, organic acids, and volatile ammonia (Li et al., 2019; Liu et al., 2015).

The ratio of potassium ion to chloride ion content (K^+^/Cl^−^) is considered to be an important indicator to measure the combustibility of tobacco leaves, with a higher ratio indicating better combustibility (Wang et al., 2021). The K^+^/Cl^−^ ratio reached its peak at 30°C or 35°C. As the temperature increased, the chloride ion content gradually rose, leading to a decrease in the K^+^/Cl^−^ ratio (Figures 1C,D). Meanwhile, the results of sensory evaluation indicated that low-temperature fermentation was more conducive to improving the combustion performance of tobacco leaves (Supplementary Table S1).

### 3.2 Comparison of flavor components

**FIGURE 2:**
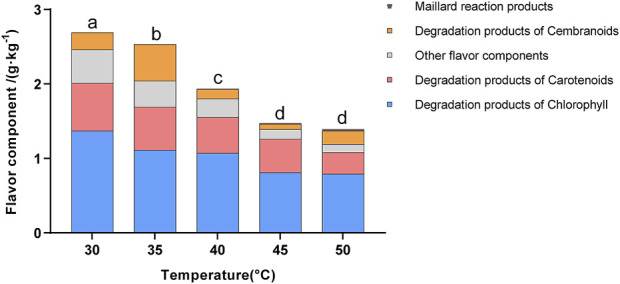
Flavor component content in tobacco leaves fermented at different fermentation temperatures. Letters indicated significant differences based on Tukey’s HSD.

Previous studies have shown that the total content of flavor components is an important indicator of the quality of cigar tobacco leaves (Banozic et al., 2021; Hu et al., 2022; Li et al., 2020). As shown in Figure 2, the highest total content of flavor components was observed at 30°C and gradually decreased with increasing temperature. Furthermore, fermentation at 30°C–35°C exhibited higher sensory scores for richness, aftertaste, and mellowness. These results indicated that fermentation at lower temperatures was more beneficial in enhancing the quantity and quality of flavor (Supplementary Table S1). This might be attributed to the fact that low-temperature fermentation promotes the growth and metabolism of microorganisms (such as yeast), thereby producing a greater variety of flavor components. A total of 41 flavor components were detected in the cigar leaves, including chlorophyll degradation products, cembranoids degradation products, carotenoid degradation products, Maillard reaction products, and other flavor compounds. According to the literature, these degradation products could enrich the flavor and are positively correlated with the maturity of tobacco leaves (Banozic et al., 2021).

**FIGURE 3:**
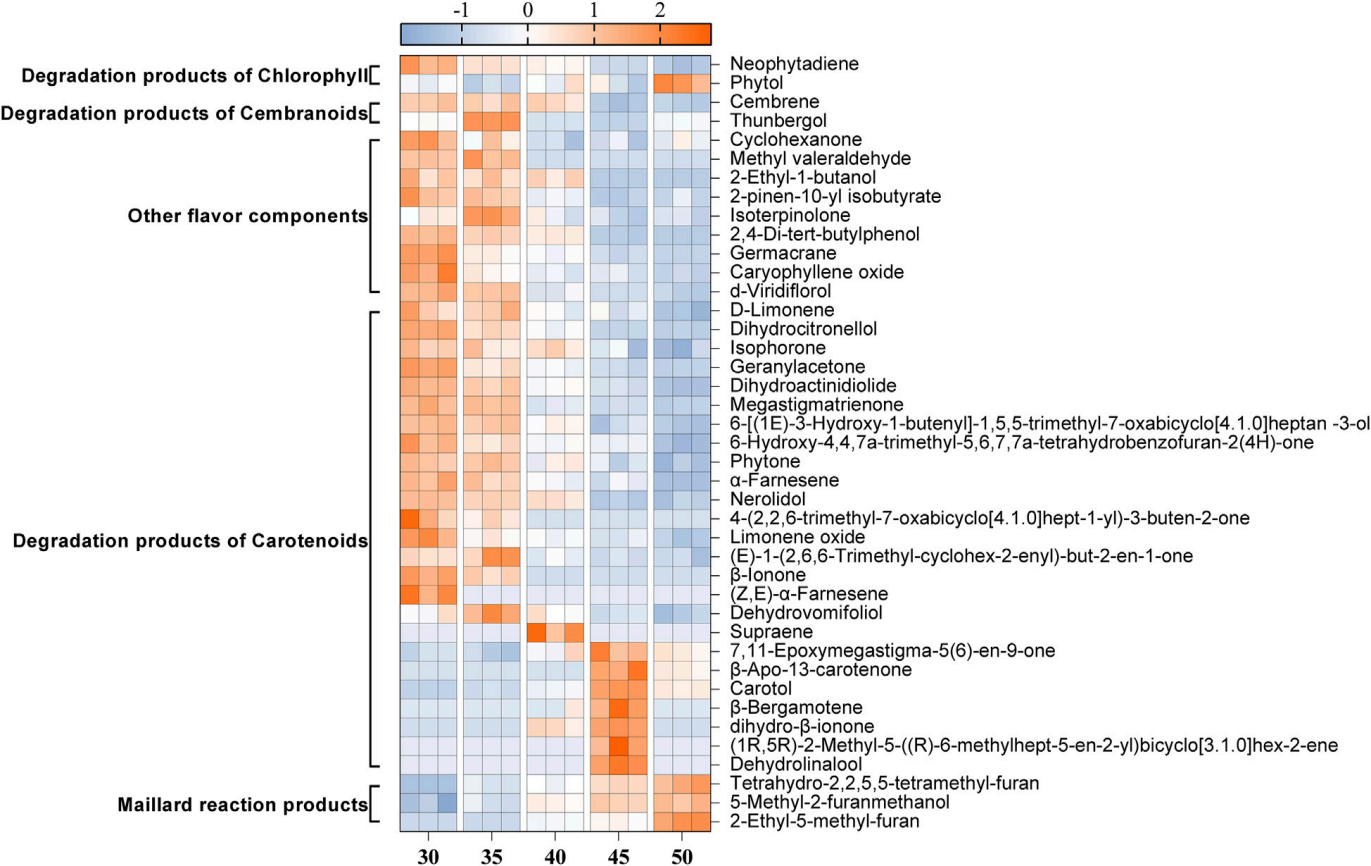
Changes in the content of 41 flavor compounds at different fermentation temperatures. The content of each flavor compound was normalized using Z-score. The color intensity was proportional to the concentration of compounds.

As depicted in Figure 3, the contents of Maillard reaction products, which contribute to caramel, nutty, and baking flavors, such as Tetrahydro-2,2,5,5-tetramethyl-furan, 5-Methyl-2-furanmethanol, and 2-Ethyl-5-methyl-furan, gradually increased with rising temperature. In contrast, the contents of chlorophyll degradation products (Neophytadiene), cembranoids degradation products (Thunbergol, Cembrene), other flavor components (2-Pinen-10-yl isobutyrate, Isoterpinolone, Germacrane), and most carotenoid degradation products (D-Limonene, Dihydrocitronellol, Isophorone, Geranylacetone, Dihydroactinidiolide, Megastigmatrienone, Phytone, α-Farnesene, Nerolidol, and β-Ionone) gradually decreased as the temperature increased. Notably, the content of Neophytadiene was the most abundant in the tobacco leaves, accounting for 42.3% of the total flavor substances. Neophytadiene has a fresh flavor that could improve the mellowness and reduce irritation (Hu et al., 2022). In this study, Thunbergol was detected as the primary degradation product of cembranoids, characterized by a cedar and herbal flavor. Furthermore, carotenoid degradation products represented the most diverse category of flavor substances found in cigar tobacco leaves. As presented in Table 1, the majority of these products exhibited strong floral, fruity and woody flavors, such as Dihydroactinidiolide and Megastigmatrienone, contributing to the unique flavor profile of cigars (Popova et al., 2019; Wang et al., 2024).

**TABLE 1:** Contents of flavor compounds of tobacco leaves after fermentation at different temperatures (mg/kg).

CAS	Compounds name	T30	T35	T40	T45	T50	Flavor^a^
504–96-1	Neophytadiene	1,199.1	967.1	889.8	649.4	554.6	Fresh
150–86-7	Phytol	170.2	146.6	180.7	159.8	236.9	Floral, balsam
Chlorophyll degradation products		1,369.2	1,113.7	1,070.5	809.2	791.5	
1898–13-1	Cembrene	40.3	39.1	36.8	14.4	16.6	Precursor
25,269–17-4	Thunbergol	194.5	447.1	98.0	50.9	166.1	Cedar, herb
Cembranoids degradation products		234.7	486.2	134.7	65.3	182.8	
7,764–50-3	Cyclohexanone	15.0	12.3	9.2	9.6	10.9	Mint
123–15-9	Methyl valeraldehyde	2.7	3.2	-	-	-	Vegetable, green, fruity
97–95-0	2-Ethyl-1-butanol	4.4	3.8	3.7	-	-	Musty, wine
29,021–37-2	2-pinen-10-yl isobutyrate	8.2	7.7	5.7	4.4	4.9	Woody, fruity, cedar
586–63-0	Isoterpinolone	10.3	14.5	8.9	7.0	7.6	Cedar
96–76-4	2,4-Di-tert-butylphenol	170.1	142.0	113.3	16.1	15.1	Fruity
645–10-3	Germacrane	23.9	10.6	7.7	1.3	-	Woody, spicy
1,139–30-6	Caryophyllene oxide	157.8	98.6	75.3	71.8	55.3	Fruity
552–02-3	d-Viridiflorol	57.5	52.4	29.4	24.4	20.1	Fruity, fresh, herb, mint, sweet
Other flavor compounds		449.9	345.2	253.3	134.6	114.0	
5,989–27-5	D-Limonene	11.7	11.7	8.0	7.4	4.1	Fruity, citrus, mint
106–21-8	Dihydrocitronellol	79.2	53.8	32.9	8.3	4.5	Floral, lemon, leather, musty
78–59-1	Isophorone	4.4	4.2	4.2	3.3	2.8	Woody, camphor, cedar, tobacco
3,796–70-1	Geranylacetone	21.0	12.8	8.1	3.9	2.5	Floral, fruity, Magnolia, fresh
15,356–74-8	Dihydroactinidiolide	32.6	28.3	19.8	13.7	7.5	Coumarin, musk, floral, fruity
38,818–55-2	Megastigmatrienone	22.7	21.1	5.9	3.6	0.8	Nutty, floral, woody
72,777–88-9	6-[(1E)-3-Hydroxy-1-butenyl]-1,5,5-trimethyl-7-oxabicyclo [4.1.0]heptan-3-ol	81.5	77.7	61.9	42.4	35.1	Floral, woody
73,410–02-3	6-Hydroxy-4,4,7a-trimethyl-5,6,7,7a-tetrahydrobenzofuran-2(4H)-one	75.2	65.2	58.3	55.9	42.9	Fruity, musk, woody
502–69-2	Phytone	68.8	68.7	58.8	51.6	44.1	Floral, herb, celery, fat
26,560–14-5	α-Farnesene	10.7	8.8	4.8	3.6	-	Fruity, herb, woody, sweet
40,716–66-3	Nerolidol	79.5	72.7	67.6	36.5	37.0	Citrus, floral, woody, cedar
23,267–57-4	4-(2,2,6-trimethyl-7-oxabicyclo [4.1.0]hept-1-yl)-3-buten-2-one	6.4	3.2	-	-	-	Fruity, woody, sweet, violet
6,909–30-4	Limoneneoxide	35.9	21.7	18.4	14.7	8.3	Fruity, sweet, fresh
24,720–09-0	(E)-1-(2,6,6-Trimethyl-cyclohex-2-enyl)-but-2-en-1-one	17.9	24.5	11.7	8.8	6.9	Floral
79–77-6	β-Ionone	11.9	7.6	-	-	-	Woody, fruity, cedar, floral
26,560–14-5	(Z,E)-α-Farnesene	4.1	-	-	-	-	Floral, woody, boiled vegetables
39,763–33-2	Dehydrovomifoliol	56.5	79.4	57.7	44.0	35.7	Floral
7,683–64-9	Supraene	-	-	10.1	-	-	Flower
64,243–62-5	7,11-Epoxymegastigma-5 (6)-en-9-one	17.3	15.5	22.3	30.2	23.5	Fruity
17,974–57-1	β-Apo-13-carotenone	-	-	-	12.0	4.6	Fruity
465–28-1	Carotol	-	5.3	19.3	70.3	33.8	Pleasant, light
1,000,425–19-8	β-Bergamotene	-	-	0.6	5.0	-	Woody, tea, warm
17,283–81-7	dihydro-β-ionone	-	-	9.1	17.3	-	Floral, woody, amber, earth
58,319–06-5	(1 R,5 R)-2-Methyl-5-((R)-6-methylhept-5-en-2-yl)bicyclo [3.1.0]hex-2-ene	-	-	-	3.7	-	Spicy, herb
29,171–20-8	Dehydrolinalool	-	-	-	11.6	-	Musty, musk
Carotenoid degradation products		637.3	582.2	479.6	447.6	294.2	
15,045–43-9	Tetrahydro-2,2,5,5-tetramethyl-furan	-	2.4	3.6	5.7	8.0	Nutty
3,857–25-8	5-Methyl-2-furanmethanol	1.6	4.4	7.1	9.1	10.3	Caramel, baking
1703–52-2	2-Ethyl-5-methyl-furan	-	-	2.6	3.8	10.2	Baking, nutty
Maillard reaction products		1.6	6.7	13.3	18.6	28.5	

^*a*^ From flavor databases (https://www.femaflavor.org/flavor-library; http://www.thegoodscentscompany.com; https://foodb.ca/compounds).

Overall, the total content of flavor components showed a gradual decline with increasing fermentation temperature, suggesting that low-temperature fermentation was beneficial to the accumulation of flavor components. Specifically, the Maillard reaction products gradually increased with rising temperature and peaked at 50°C, indicating that high-temperature fermentation enhanced the baking and nutty flavors in cigar tobacco leaves (Alsafra et al., 2023). Conversely, the contents of carotenoid and chlorophyll degradation products, as well as other flavor components, gradually decreased with increasing temperature and were highest at 30°C. This indicated that low-temperature fermentation was beneficial for enriching the flavor complexity of cigar leaves, such as floral, fruity, and woody flavors.

### 3.3 Effect of temperature on biomass

**FIGURE 4:**
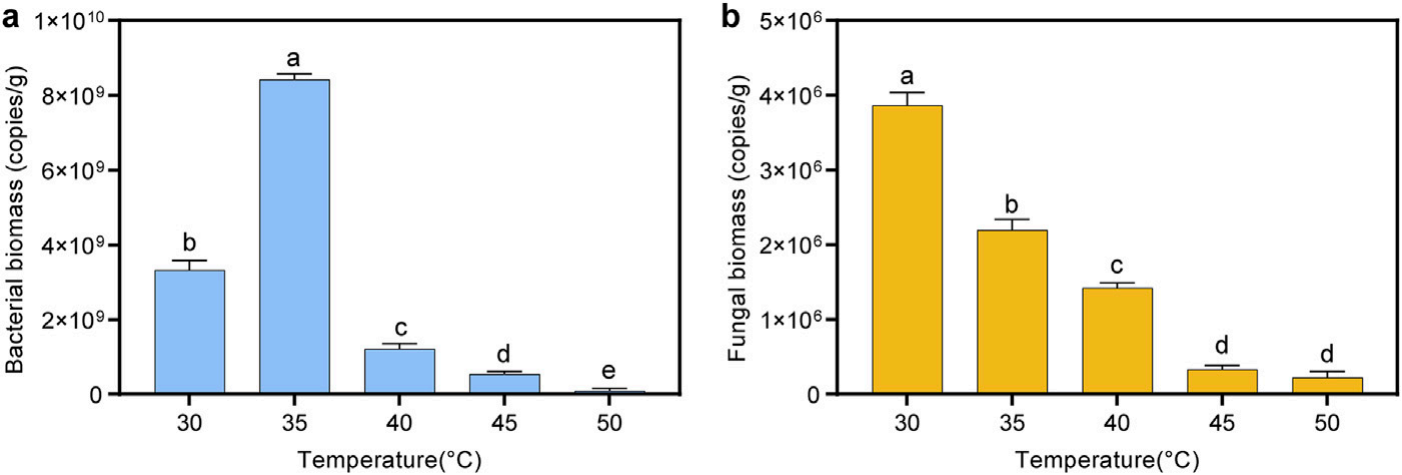
Changes in bacterial and fungal biomass in cigar tobacco leaves under different fermentation temperatures. **(A)** Bacterial biomass; **(B)** fungal biomass.

In this study, the effects of different fermentation temperatures on the bacterial and fungal biomass in cigar tobacco leaves were investigated using quantitative real-time PCR. As shown in Figure 4, the bacterial biomass initially increased and then decreased with increasing temperature, peaking at 8.4 × 10^9^ copies/g at 35°C. This trend might be attributed to the optimal growth temperature for most bacteria, which is around 35°C. When the fermentation temperature exceeded 40°C, bacterial growth was significantly inhibited, and the biomass decreased to 8.6 × 10^7^ copies/g at 50°C. In addition, the fungal biomass exhibited a consistent decline with increasing temperature, with the highest biomass observed at 30°C, reaching 3.9 × 10^6^ copies/g. This indicated that the optimal temperature for fungal community growth in cigar tobacco leaves was approximately 30°C. When the fermentation temperature exceeded 45°C, the growth of the fungal community was significantly inhibited, resulting in a biomass of only 2.2 × 10^5^–3.3 × 10^5^ copies/g.

### 3.4 Changes in microbial diversity

**FIGURE 5:**
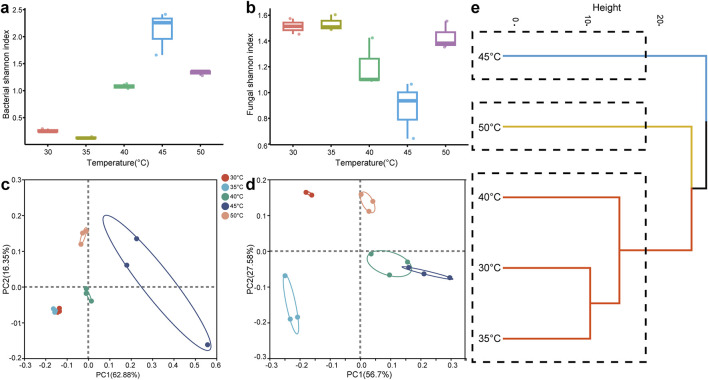
Microbial diversity analysis. **(A)** Bacterial Shannon-Wiener index; **(B)** fungal Shannon-Wiener index; **(C)** PCoA analysis of bacterial community; **(D)** PCoA analysis of fungal community; **(E)** clustering analysis of microbial community.

Exploring microbial diversity across various conditions is crucial for understanding how microbial communities adapt to environmental changes. As shown in Figure 5A, the Shannon index for bacterial diversity first rose and then fell with increasing temperature, peaking at 45°C, indicating that bacterial diversity was greatest at this temperature. Conversely, the Shannon index for fungal diversity initially declined and then increased as the temperature rose (Figure 5B). The PCoA analysis revealed that the bacterial community structure was similar under the fermentation conditions of 30°C and 35°C (Figure 5C). However, as the temperature increased, bacterial community structure changed significantly. Similarly, fungal community structure also experienced substantial alterations with increasing temperature (Figure 5D). Based on the clustering analysis of bacterial and fungal communities in Figure 5E, the samples under different temperature conditions were classified into three groups: a low-temperature group (30°C, 35°C, and 40°C), a medium-temperature group (45°C) and a high-temperature group (50°C). These results demonstrated that temperature changes had a significant impact on the diversity of microbial communities and played a key role in shaping distinct microbial community structures.

### 3.5 Analysis of microbial community structure

**FIGURE 6:**
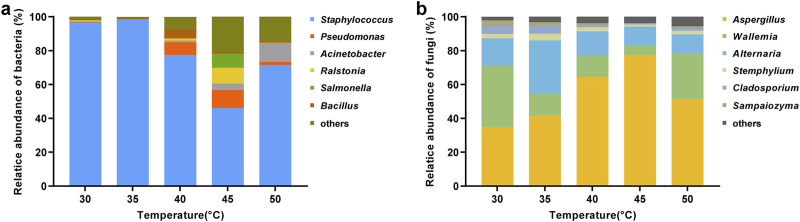
Changes in microbial community structure in cigar leaves at the genus level under different fermentation temperatures. **(A)** Bacteria; **(B)** fungi. The heights of different colors represented the relative abundance of different microbial genera in the overall bacterial or fungal community.

Generally, dominant microbial genera with high abundance (average relative abundance >1%) are considered to play an important role in the fermentation process (Jia et al., 2020). Figure 6A revealed that *Staphylococcus* (78.1%), *Pseudomonas* (4.0%), *Acinetobacter* (3.2%), *Ralstonia* (2.3%), *Salmonella* (1.7%), and *Bacillus* (1.5%) were the dominant bacterial genera in cigar leaves. *Staphylococcus* occupied the dominant position in the bacterial community, and its relative abundance initially decreased and then increased with rising temperature, reaching a maximum of 98.5% at 35°C and a minimum of 46.1% at 45°C. In contrast, the relative abundance of *Pseudomonas*, *Ralstonia*, *Salmonella*, and *Bacillus* genera increased first and then declined. Among them, *Bacillus* reached its highest relative abundance at 40°C (5.3%), and *Pseudomonas*, *Ralstonia*, and *Salmonella* peaked at 45°C with respective abundances of 10.6%, 9.4%, and 8.4%. Additionally, the relative abundance of *Acinetobacter* increased with increasing temperature, peaking at 11.2% at 50°C.

As shown in Figure 6B, dominant fungal genera found in cigar leaves were primarily *Aspergillus* (54.0%), *Wallemia* (18.8%), *Alternaria* (16.9%), *Stephylium* (2.5%), *Cladosporium* (2.5%), and *Sampaiozyma* (1.6%). *Aspergillus* was the predominant genus in the fungal community, and its relative abundance first increased and then declined with rising temperature, peaking at 77.4% at 45°C and decreasing to 34.9% at 30°C. *Alternaria* and *Stephylium* also showed an initial increase followed by a decrease with temperature, reaching their peaks at 35°C with respective abundances of 31.4% and 4.1%. In contrast, the relative abundance of *Wallemia*, *Cladosporium*, and *Sampaiozyma* genera first decreased and then increased, reaching their highest values at 30°C, at 36.0%, 4.9%, and 3.2%, respectively.

**FIGURE 7:**
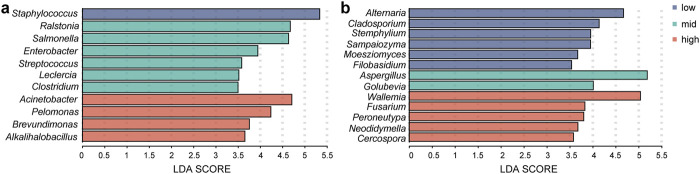
Identification of microbial biomarkers in the low-temperature, medium-temperature, and high-temperature fermentation groups. **(A)** Bacteria; **(B)** fungi.

In this study, LEfSe analysis was conducted with a threshold LDA score of 3.0 to identify biomarkers under different fermentation temperatures. Figure 7 illustrated the biomarkers found in each fermentation group. At low temperature, bacterial biomarkers were represented by *Staphylococcus*, and fungal biomarkers included *Alternaria*, *Cladosporium*, *Stemphylium*, *Sampaiozyma*, *Moesziomyces*, and *Filobasidium*. For the medium-temperature fermentation group, bacterial biomarkers included *Ralstonia*, *Salmonella*, *Enterobacter*, *Stenotrophomonas*, *Leclercia*, and *Clostridium*, and fungal biomarkers were *Aspergillus* and *Golubevia*. In the high-temperature fermentation group, the bacterial biomarkers consisted of *Acinetobacter*, *Pelomonas*, *Brevundimonas*, and *Alkalihalobacillus*, fungal biomarkers included *Wallemia*, *Fusarium*, *Peroneutypa*, *Neodidymella*, and *Cercospora*. The findings suggested that genera such as *Acinetobacter*, *Pelomonas*, *Wallemia*, and *Aspergillus* exhibited strong tolerance to high temperature and might play pivotal roles in the high-temperature fermentation process of tobacco leaves.

### 3.6 Prediction of microbial function

**FIGURE 8:**
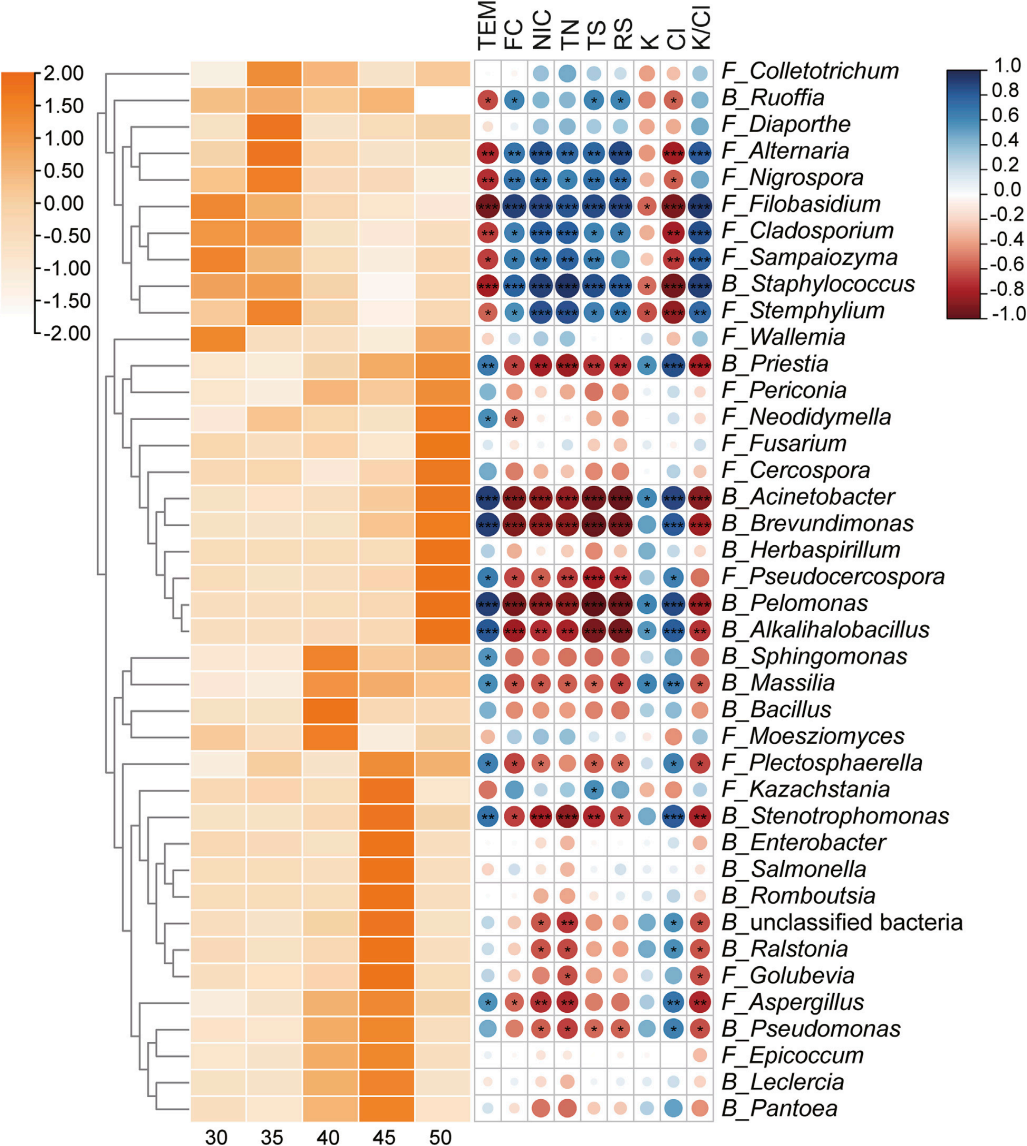
Succession of the Top 20 genera in bacterial and fungal communities, respectively, and their correlations with fermentation indicators. The relative abundance of each microbial genus was normalized using Z-score. The succession of microbial genera was presented on the left, with color intensity positively associated with relative abundance. The right side showed the Spearman correlation between each genus and fermentation indicators, the correlation coefficient was represented by the color and size of the circles, dark blue for positive correlation and dark red for negative correlation. TEM: temperature; TS: total sugar; RS: reducing sugar; TN: total nitrogen; NIC: alkaloid. Statistical significance was denoted by *P < 0.05; **P < 0.01; ***P < 0.001.

This study not only investigated the changes in microbial communities with varying fermentation temperatures but also analyzed the functional potential of microbial communities. Spearman correlation coefficients were calculated to assess the relationship between microbial genera and physicochemical indicators. Figure 8 revealed that certain microbial genera (*Ruoffia*, *Alternaria*, *Nigrospora*, *Filobasidium*, *Cladosporium*, *Sampaiozyma*, *Staphylococcus*, and *Stemphylium*) that were negatively correlated with temperature were significantly positively correlated with the content of flavor substances, total nitrogen, alkaloids, total sugar, reducing sugar and the K^+^/Cl^−^ ratio, while negatively correlated with potassium ion and chloride ion content. The results indicated that these microbial genera might play a significant role in promoting the formation of flavor substances, the accumulation of sugars, and the increase of the K^+^/Cl^−^ ratio. Instead, microbial genera (*Priestia*, *Acinetobacter*, *Brevundimonas*, *Pseudocercospora*, *Pelomonas*, *Alkalihalobacillus*, *Sphingomonas*, *Massilia*, *Plectosphaerella*, *Stenotrophomonas*, and *Aspergillus*) that were positively correlate with temperature exhibited a significant negative correlation with the contents of flavor compounds, total nitrogen, alkaloids, total sugars, reducing sugars, and the K^+^/Cl^−^ ratio. Conversely, they displayed a positive correlation with the concentrations of potassium and chloride ions. These observations suggested that these microorganisms might contribute to the degradation of nitrogen-containing substances and alkaloids.

**FIGURE 9:**
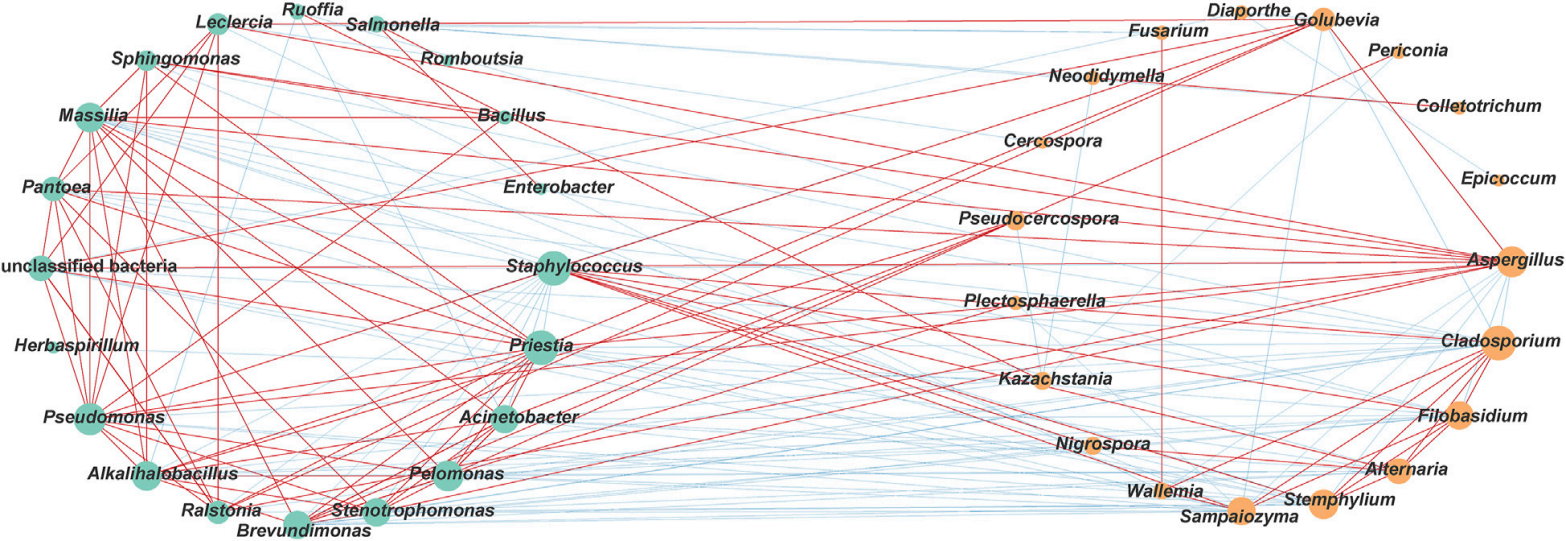
Correlation network of co-occurrence and co-exclusion relationships between different genera. Statistically significant (*P* < 0.05) Spearman’s correlation coefficient (|*ρ*| > 0.6) indicates the robust correlations. The size of nodes indicates the degree of connections. Green and orange nodes indicate bacteria and fungi respectively. Edge thickness represents the proportional to the value of Spearman’s correlation. Bule and red edges indicate negative and positive interaction between nodes.

In addition, co-occurring taxa could have a significant impact on the structure and function of microbial communities (Banerjee et al., 2018). As shown in Figure 9, 175 pairs of significant and robust edges were obtained (90 positive correlations and 85 negative correlations) (|*ρ*| > 0.6, *P* < 0.05) based on microbial network analysis. Among them, there were 26 pairs of positive correlation and 58 pairs of negative correlation between bacterial and fungal genera, and the negative correlations accounted for 69.0%. In particular, there was a significant negative correlation between the dominant fungus *Aspergillus* and the dominant bacterium *Staphylococcus* (*ρ* = −0.78). These results indicated that there might be a competitive relationship between bacterial and fungal communities. In addition, 49 pairs of positive and 13 pairs of negative correlations were found within the bacterial microbial community, while 15 pairs of positive and 14 pairs of negative correlations were found within the fungal community. This indicated that there might be a cooperative or synergistic relationship within bacterial communities. In this study, nodes with degrees exceeding 10 were defined as co-occurring taxa. As shown in Supplementary Tables S4, S7 fungal genera (*Cladosporium*, *Aspergillus*, *Stemphylium*, *Filobasidium*, *Sampaiozyma*, *Alternaria*, and *Golubevia*) and 11 bacterial genera (*Staphylococcus*, *Priestia*, *Pseudomonas*, *Pelomonas*, *Alkalihalobacillus*, *Massilia*, *Acinetobacter*, *Stenotrophomonas*, *Brevundimonas*, *Pantoea*, and *Ralstonia*) were identified as co-occurring taxa.

## 4 Discussion

The fermentation temperature had a significant impact on the chemical composition and flavor quality of tobacco leaves. The study revealed that low-temperature fermentation (30°C–35°C) was conducive to the accumulation of sugars and flavor components (carotenoid degradation products, chlorophyll degradation products, and other flavor components), as well as the increase of the K^+^/Cl^−^ ratio. This might be because the low temperatures were conducive to the growth of microorganisms (e.g., yeast) closely related to the generation of flavor substances and sugar compounds. For example, Filobasidium enriched at low temperatures could not only secrete α-amylase to produce sugars from carbohydrates, but also secrete lipoxygenase to produce flavor substances (Mot and Verachtert, 1985; Wu, 2022). Conversely, high-temperature fermentation (45°C–50°C) promoted the generation of Maillard reaction products and the degradation of total nitrogen and alkaloids. This might be because the medium-high temperature could promote the growth of enzyme-producing microorganisms (e.g., *Alkalihalobacillus*) and the action of enzyme activity, leading to the degradation of proteins and alkaloids into smaller molecules (Dilmaghani and Far, 2020; Li et al., 2019; Liu et al., 2015). For example, the spore preparations of *Alkalihalobacillus clausii* could remain stable under strong acid and high-temperature conditions, and have been applied to the digestion of proteins in functional foods. In addition, fermentation at higher temperatures exacerbated the Maillard reaction, resulting in the reduction of sugar content and the formation of Maillard reaction products (Alsafra et al., 2023). According to the literature, the flavor richness and maturity of tobacco leaves were directly related to the content and types of flavor components (Banozic et al., 2021; Li et al., 2020). When the protein and alkaloid content in tobacco leaves was too high, it resulted in bitterness, astringency, and strong irritancy in tobacco leaves (Kaminski et al., 2020; Liu et al., 2021). In addition, a higher ratio of potassium ions to chloride ions in tobacco leaves typically indicated better combustion performance (Wang et al., 2021). Therefore, low-temperature fermentation could improve combustibility and flavor richness, while high-temperature fermentation could significantly reduce irritancy and improve the cleanliness of tobacco leaves. By controlling the appropriate temperature at different fermentation stages, the flavor components content and flavor richness could be significantly improved while reducing the alkaloid content and irritancy. Researchers have used variable temperature fermentation technology to improve the yield and quality of fermented products such as soybean paste, tea, and cigars (Huang et al., 2022; Jia et al., 2023c; Zhao, 2011).

Fermentation temperature is a key environmental factor that regulates microbial growth and metabolism. This study investigated the effects of different temperatures on the microbial community of cigar tobacco leaves through qPCR and high-throughput sequencing. The findings revealed that the biomass of bacteria and fungi were the highest at 30°C–35°C, and the growth of both was significantly inhibited when fermentation temperature exceeded 45°C. Additionally, as the fermentation temperature increased, the structure of microbial community changed significantly. Through clustering analysis, the treatments at different temperatures were categorized into three groups: the low-temperature group (30°C, 35°C, and 40°C), the medium-temperature group (45°C), and the high-temperature group (50°C). The dominant genera within the bacterial community were primarily *Staphylococcus*, *Pseudomonas*, *Acinetobacter*, *Ralstonia*, *Salmonella*, and *Bacillus*, while the fungal community was mainly composed of *Aspergillus*, *Wallemia*, *Alternaria*, *Stemphylium*, *Cladosporium*, and *Sampaiozyma*. *Staphylococcus* and *Aspergillus* genera dominated the bacterial and fungal communities, respectively. As the temperature increased, the relative abundance of *Staphylococcus* initially declined and then increased (46.1%–98.5%), whereas the relative abundance of *Aspergillus* followed an opposite trend, increasing first and then decreasing (34.9%–77.4%). Furthermore, the Shannon index of bacteria first increased and then decreased with the rise of temperature, reaching its peak at 45°C. In contrast, the Shannon index of fungi decreased first and then increased, which was opposite to that of bacteria. This shift in diversity within microbial communities might result from the interplay of temperature fluctuations and interactions among the microorganisms (Ren et al., 2023). At elevated temperatures, although the total biomass of microorganisms was significantly inhibited, certain microbial genera were capable of adapting and surviving, enabling the entire community to adapt to the new environmental conditions. For example, at a fermentation temperature of 45°C, the relative abundance of the dominant genus *Staphylococcus* was the lowest, and other bacteria that could adapt to the high-temperature environment (such as *Acinetobacter*, *Pseudomonas*, *Ralstonia*, *Salmonella*, *Bacillus*) seized the opportunity to grow and reproduce, resulting in a significant increase in the alpha diversity of bacteria. Studies have shown that when dominant microorganisms proliferate in large numbers, the diversity of microbial communities tends to decline, which might be attributed to the antagonism among microorganisms (Coburn et al., 2015).

To further reveal how microbial communities affect the quality changes in tobacco leaves, this study explored the correlation between microorganisms and physicochemical metabolites. The results indicated that under low-temperature fermentation conditions, specific microbial populations, including *Staphylococcus*, *Alternaria*, *Cladosporium*, *Sampaiozyma*, *Filobasidium*, etc., promoted the increase of flavor substances and sugar compounds, as well as the elevation in the K^+^/Cl^−^ ratio. Conversely, in medium and high-temperature fermentation environments, microorganisms such as *Acinetobacter*, *Brevundimonas*, *Pelomonas*, *Alkalihalobacillus*, *Aspergillus*, etc., were more effective in degrading nitrogen-containing substances and alkaloids. It has been reported that *Staphylococcus*, *Sampaiozyma,* and *Filobasidium* were closely associated with the production of flavor substances and were used in the fermentation of foods like soy sauce and wine (Merín et al., 2013; Wang et al., 2024; Zhang et al., 2024). For instance, *Filobasidium* could produce high levels of lipoxygenase, significantly improving the flavor quality of tobacco leaves (Wu, 2022). *Alternaria* and *Cladosporium* were prevalent pathogens causing brown spots in tobacco leaves and could cause damage to agricultural crops (Wang et al., 2014; Xiang et al., 2022). In addition, *Aspergillus*, *Alkalihalobacillus*, and *Brevundimonas* were known to secrete proteases that effectively degraded macromolecular substances, and they have been widely used in the processing of soy sauce, feed, meat, and textiles (Nadeem et al., 2024; Olivera et al., 2020; Rehbar and Batool, 2017). *Acinetobacter* and *Pelomonas* have been reported to have denitrification ability and exhibit significant potential for nitrogen removal in wastewater treatment (Wu et al., 2024; Zhong and Xia, 2023). In addition, microbial network analysis indicated that seven fungal genera (*Cladosporium*, *Aspergillus*, *Stemphylium*, *Filobasidium*, *Sampaiozyma*, *Alternaria*, *Golubevia*) and 11 bacterial genera (*Staphylococcus*, *Priestia*, *Pseudomonas*, *Pelomonas*, *Alkalihalobacillus*, *Massilia*, *Acinetobacter*, *Stenotrophomonas*, *Brevundimonas*, *Pantoea*, *Ralstonia*) were identified as co-occurring taxa. They emphasized the importance of numerically insignificant genera in maintaining the structural and functional stability of the microbial community during cigar tobacco fermentation (Wu et al., 2019). For example, *Filobasidium* might play a crucial role in complex fermentation systems, despite accounting for only 0.1% of the total fungal abundance. In addition, the prevalent negative correlation between bacterial and fungal communities suggested that there might be a competitive relationship between bacterial and fungal communities. Overall, environmental factors and microbial interactions collectively contribute to the distribution and function of microbial communities. However, the lag effect of microbial metabolic activity and the complexity of environmental factors can lead to inconsistencies between microbial succession and metabolites dynamics, resulting in erroneous causal relationships. Therefore, it is necessary to further verify the functional characteristics of microorganisms through *in vitro* simulated fermentation experiments.

## 5 Conclusion

In this study, Dexue one was used as the research subject to explore the effects of different fermentation temperatures on the structure and function of microbial communities. When the temperature exceeded 45°C, the growth of microorganisms was significantly inhibited. The microbial communities (*Staphylococcus*, *Sampaiozyma,* and *Filobasidium*) that developed under low-temperature conditions were more conducive to the formation of flavor substances, the accumulation of sugars, and the improvement of the K^+^/Cl^−^ ratio, to improve the flammability and flavor richness. In contrast, microbial communities (*Acinetobacter*, *Brevundimonas*, *Pelomonas*, *Alkalihalobacillus*, and *Aspergillus*) formed under high-temperature conditions showed greater capacity for degrading nitrogen-containing substances and alkaloids, contributing to reduced irritation and improved cleanliness. These results indicated that fermentation temperatures could affect microbial growth and shape unique microbial community structures, which further affected the physicochemical metabolites and final quality of cigar tobacco leaves. The basic principles of microbial community function and their response to fermentation temperature are essential for further optimizing the fermentation process of cigar tobacco leaves and developing functional microorganisms adapted to different fermentation temperatures. However, due to variations in origin, year, variety, grade, and maturity of tobacco leaves, there were differences in the chemical composition and required fermentation conditions for different types of tobacco leaves. Therefore, parameters such as fermentation temperature need to be adjusted based on the specific characteristics of the tobacco varieties. In the future, metagenomic analysis will be employed to assess the contribution of microorganisms to specific metabolic pathways, and the metabolic functions of potential functional microorganisms will be isolated and validated.

## Data Availability

The raw sequence data reported in this paper have been deposited in the Genome Sequence Archive at the Beijing Institute of Genomics (BIG) Data Center, Chinese Academy of Sciences, under accession numbers PRJCA034066 were publicly accessible at https://bigd.big.ac.cn/gsa.
