# Supplementary material for: Effects of different fermentation temperatures on microbiomes of cigar tobacco leaves
Source: Front Bioeng Biotechnol. 2025 Feb 25;13:1550383. doi: 10.3389/fbioe.2025.1550383 (PMC11893599; doi:10.3389/fbioe.2025.1550383)
Supplement: Supplementary file 1 [file DataSheet1.pdf]

Supplementary Material

Effects of Different Fermentation Temperatures on Microbiomes of Cigar Tobacco Leaves

Yun Jia[1,2], Sida Guo[1], Wanrong Hu[1], Qianying Zhang[1], Yue Wang[1], Zhengcheng Zhang[1], Zhishun Chai[1], Dongliang Li[1,*]

[1] China Tobacco Technology Innovation Center for Cigar, China Tobacco Sichuan Industrial Co., Ltd., Chengdu 610100, Sichuan, China

[2]Industry Efficient Utilization to Domestic Cigar Tobacco Key Laboratory of Sichuan Province, China Tobacco Sichuan Industrial Co., Ltd., Shifang 618400, Sichuan, China

*** Correspondence**

Dongliang Li: 360188228@qq.com

1 Supplementary Figures

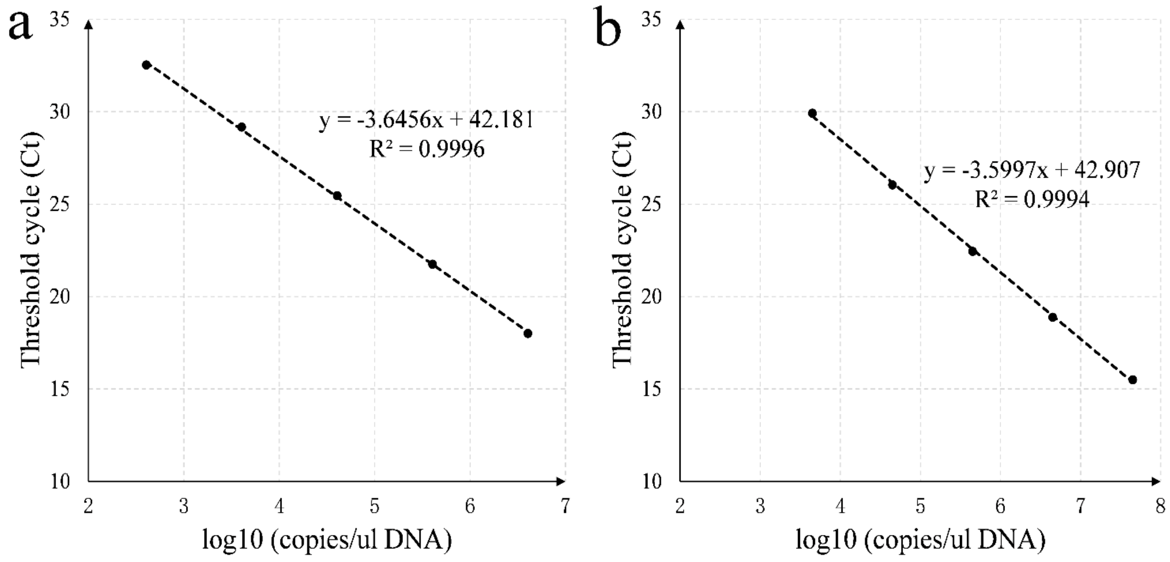

Supplementary Figures 1 The standard curves for quantifying the bacteria (a) and fungi (b)

Supplementary Table 1 Sensory evaluation of cigar tobacco leaves at different fermentation temperatures

Temperature / Characteristics	30 ℃	35 ℃	40 ℃	45 ℃	50 ℃
mellowness	6.0	6.5	5.5	5.5	4.0
richness	6.5	6.5	5.5	5.0	4.5
matureness	6.0	6.5	6.0	6.0	5.5
irritation	5.0	5.0	5.5	6.0	6.0
smoothness	6.0	6.0	5.5	5.5	5.5
fluentness	6.0	6.0	5.5	5.5	5.5
sweetness	6.0	6.5	5.0	4.5	4.0
cleanliness	5.0	5.5	6.0	6.5	6.5
aftertaste	6.5	6.5	6.0	5.5	5.0
combustibility	6.5	6.5	6.0	6.0	6.0
ash color	6.5	6.5	6.5	6.5	6.5
ash coagulation	6.5	6.5	6.5	6.5	6.5

Supplementary Table 2 Relative abundance of bacterial genera at different temperature (%)*

Temperature / Genera	30℃	35℃	40℃	45℃	50℃
Staphylococcus	96.6±0.4^{ab}	98.5±0.2^{a}	77.5±0.0^{bc}	46.1±17.3^{d}	71.6±1.4^{c}
Pseudomonas	0.4±0.3^{a}	0.1±0.1^{a}	7.4±0.6^{a}	10.6±9.7^{a}	1.7±0.5^{a}
Acinetobacter	0.1±0.1^{b}	0.0±0.0^{b}	1.0±0.1^{b}	3.7±0.8^{b}	11.2±3.7^{a}
Ralstonia	0.8±0.4^{b}	0.0±0.0^{b}	1.2±0.3^{b}	9.4±6.0^{a}	0.1±0.0^{b}
Salmonella	0.1±0^{a}	0.1±0.1^{a}	0.0±0.0^{a}	8.4±13.8^{a}	0.0±0.0^{a}
Bacillus	0.3±0.2^{b}	0.2±0.2^{b}	5.3±1.1^{a}	0.9±0.5^{b}	0.7±0.3^{b}
others	1.8±0.2±^{c}	1.0±0.2^{c}	7.5±1.1^{bc}	21.0±9.5^{a}	14.7±2.8^{ab}

* Letters indicated significant differences based on Tukey's HSD.

Supplementary Table 3 Relative abundance of fungal genera at different temperature (%)*

Genera \ Temperature	30℃	35℃	40℃	45℃	50℃
Aspergillus	34.9±1.0[d]	41.6±6.3[cd]	64.6±7.9[ab]	77.4±7.9[a]	51.3±4.0[bc]
Wallemia	36.0±1.0[a]	12.8±3.3[c]	12.5±1.8[c]	5.9±2.9[d]	26.6±1.3[b]
Alternaria	16.3±0.2[b]	31.4±2.9[a]	14.3±3.1[b]	10.9±3.9[b]	11.6±1.7[b]
Stemphylium	2.6±0.2[b]	4.1±0.4[a]	2.5±0.5[b]	1.2±0.3[c]	2.2±0.8[bc]
Cladosporium	4.9±0.9[a]	4.5±1.1[a]	1.1±0.4[b]	0.4±0.2[b]	1.4±0.1[b]
Sampaiozyma	3.2±0.6[a]	2.2±0.6[ab]	1.1±0.3[bc]	0.4±0.0[c]	1.3±0.6[bc]
others	2.2±0.5[a]	3.3±0.3[a]	3.9±2.0[a]	3.8±1.2[a]	5.6±1.6[a]

* Letters indicated significant differences based on Tukey's HSD.

Supplementary Table 4 Co-occurring genera*[a, b]*

Genera	Kingdom	Degree	Relative abundance
Cladosporium	Fungi	19	2.4%
Staphylococcus	Bacteria	19	78.1%
Priestia	Bacteria	19	0.2%
Pseudomonas	Bacteria	17	4.0%
Aspergillus	Fungi	16	54.0%
Pelomonas	Bacteria	16	0.8%
Stemphylium	Fungi	15	2.5%
Alkalihalobacillus	Bacteria	15	0.2%
Massilia	Bacteria	15	0.2%
Acinetobacter	Bacteria	14	3.2%
Filobasidium	Fungi	14	0.1%
Stenotrophomonas	Bacteria	14	0.2%
Brevundimonas	Bacteria	14	0.3%
Sampaiozyma	Fungi	14	1.7%
Alternaria	Fungi	12	16.7%
Pantoea	Bacteria	11	0.2%
Ralstonia	Bacteria	10	2.3%
Golubevia	Fungi	10	0.7%

[a] The number represented the number of genera coexisting with other genera.
[b] Genera with degree above 10 were defined as co-occurring genera.
